# Structural and functional characterization of human and murine C5a anaphylatoxins

**DOI:** 10.1107/S139900471400844X

**Published:** 2014-05-30

**Authors:** Janus Asbjørn Schatz-Jakobsen, Laure Yatime, Casper Larsen, Steen Vang Petersen, Andreas Klos, Gregers Rom Andersen

**Affiliations:** a Department of Molecular Biology and Genetics, Aarhus University, Gustav Wieds Vej 10C, DK-8000 Aarhus, Denmark; b Department of Biomedicine, Aarhus University, Bartholin Building, Wilhelm Meyers Allé 4, DK-8000 Aarhus, Denmark; c Institute for Medical Microbiology and Hospital Epidemiology, Medical School Hannover, Hannover, Germany

**Keywords:** complement anaphylatoxins, C5a, C5a-desArg, GPCR activation, three-helix bundle

## Abstract

Complement is an ancient part of the innate immune system that plays a pivotal role in protection against invading pathogens and helps to clear apoptotic and necrotic cells. Upon complement activation, a cascade of proteolytic events generates the complement effectors, including the anaphylatoxins C3a and C5a. Signalling through their cognate G-protein coupled receptors, C3aR and C5aR, leads to a wide range of biological events promoting inflammation at the site of complement activation. The function of anaphylatoxins is regulated by circulating carboxypeptidases that remove their C-terminal arginine residue, yielding C3a-desArg and C5a-desArg. Whereas human C3a and C3a-desArg adopt a canonical four-helix bundle fold, the conformation of human C5a-desArg has recently been described as a three-helix bundle. Here, the crystal structures of an antagonist version of human C5a, A8^Δ71–73^, and of murine C5a and C5a-desArg are reported. Whereas A8^Δ71–73^ adopts a three-helix bundle conformation similar to human C5a-desArg, the two murine proteins form a four-helix bundle. A cell-based functional assay reveals that murine C5a-desArg, in contrast to its human counterpart, exerts the same level of activition as murine C5a on its cognate receptor. The role of the different C5a conformations is discussed in relation to the differential activation of C5a receptors across species.

## Abstract 2

The structure of the human C5aR antagonist, C5a-A8, reveals a three-helix bundle conformation similar to that observed for human C5a-desArg, whereas murine C5a and C5a-desArg both form the canonical four-helix bundle. These conformational differences are discussed in light of the differential C5aR activation properties observed for the human and murine complement anaphylatoxins across species.

## 1. Introduction   

Complement is a central component of innate immunity, acting as a first line of defence against invading pathogens and maintaining homeostasis (Walport, 2001 ▶; Ricklin *et al.*, 2010 ▶). When danger originating from non-self (pathogens) or altered-self (*e.g.* apoptotic or necrotic cells) is detected, complement initiates a complex biochemical cascade that involves more than 50 proteins ranging from soluble plasma factors to membrane-bound receptors. This cascade enables the proteolytic cleavage of the central complement components C3, C4 and C5, yielding the two opsonins C3b and C4b and the effector of the complement terminal pathway, C5b, that triggers formation of the lytic membrane attack complex (MAC; Walport, 2001 ▶; Dunkelberger & Song, 2010 ▶; Ricklin *et al.*, 2010 ▶; Carroll & Sim, 2011 ▶). Cleavage-dependent activation of C3, C4 and C5 also releases into the serum the three 10 kDa proteins C3a, C4a and C5a, known as complement anaphylatoxins. While the function of C4a remains unclear in the absence of identified C4a receptors, C3a and C5a are well characterized pro-inflammatory mediators (Hugli, 1990 ▶; Guo & Ward, 2005 ▶; Klos *et al.*, 2009 ▶). Their function in inflammation is mediated by their signalling through cognate heptahelical receptors on the host cells: C3aR for C3a, and C5aR and C5L2 for C5a (Klos *et al.*, 2013 ▶). Anaphylatoxin signalling through the G-protein coupled receptors (GPCRs) C3aR and C5aR leads to various cellular responses including chemotaxis, oxidative burst, the release of pro-inflammatory molecules and activation of the adaptive immune system (Klos *et al.*, 2009 ▶; Zhou, 2012 ▶). Owing to the lack of binding motifs enabling its coupling to G proteins, C5L2 has long been considered as a decoy receptor. However, recent reports now suggest that it actively participates in orchestrating pro-inflammatory events (Li *et al.*, 2013 ▶) and the involvement of C5L2 in regulating metabolism in adipose tissue has also been proposed (MacLaren *et al.*, 2008 ▶; Klos *et al.*, 2013 ▶).

**Figure 1:**
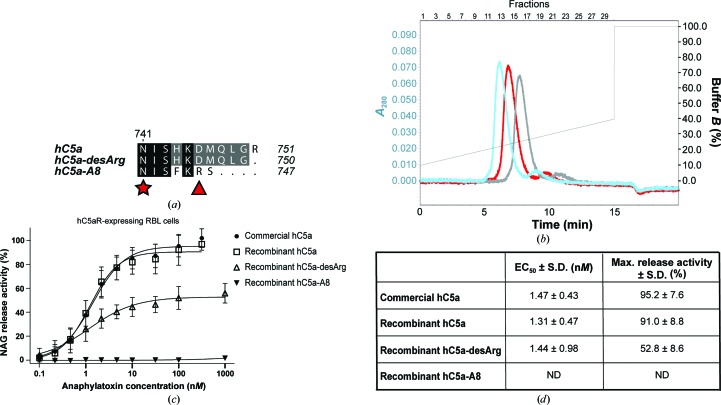
Biological activity of recombinant hC5a-derived anaphylatoxins. (*a*) Amino-acid sequence of the C-termini of hC5a, hC5a-desArg and hC5a-A8. The positions of the glycosylated Asn (star) and of the residue conditioning the agonist/antagonist shift for C5a proteins (triangle) are indicated. (*b*) Elution profile of recombinant hC5a (blue), hC5a-desArg (red) and hC5a-A8 (grey) on the SOURCE 15S column. (*c*) GARA for recombinant hC5a, hC5a-desArg and hC5a-A8 on stably transfected RBL cells expressing hC5aR and comparison with commercially available hC5a. All results are indicated as the means ± standard deviation of at least eight individual experiments (except for hC5a-A8, where *n* = 4). The NAG release activity is expressed as a percentage of the maximal NAG release activity obtained with commercially available recombinant hC5a. (*d*) EC_50_ and maximal NAG release activity observed for all proteins upon incubation with hC5aR-expressing RBL cells.

C5a is the most potent pro-inflammatory mediator among complement proteins. It targets a broad range of immune cells including basophils, neutrophils, eosinophils, mast cells and macrophages, as well as cells from nonmyeloid lineage, notably in the lung and liver (Guo & Ward, 2005 ▶) and lymphocytes, in particular regulatory T-cells (Strainic *et al.*, 2013 ▶; van der Touw *et al.*, 2013 ▶; Kemper & Köhl, 2013 ▶). As for C3a, C5a activity is regulated by carboxypeptidases that cleave off the C-terminal arginine residue (Bokisch & Müller-Eberhard, 1970 ▶), producing the C5a-desArg molecule which has a distinct bioactivity pattern and generally a much lower potency than C5a, in part owing to its tenfold to 100-fold decrease in C5aR binding affinity (Bürgi *et al.*, 1994 ▶; Eglite *et al.*, 2000 ▶; Higginbottom *et al.*, 2005 ▶). Elevated C5a plasma levels correlate with the onset of various inflammatory disorders including sepsis, rheumatoid arthritis, acute lung injury, ischaemia-reperfusion injury, allergy and asthma (Guo & Ward, 2005 ▶; Klos *et al.*, 2009 ▶, 2013 ▶), implicating C5a as a causative or exacerbating agent in the pathogenesis of these conditions. Increasing evidence also suggests a direct role of C5a and C5aR signalling in tumour progression (Markiewski *et al.*, 2008 ▶). As a consequence, targeting of the C5a–C5aR axis is considered to be a promising therapeutical strategy to downregulate complement-mediated inflammation while preserving primary defence functions such as opsonization and MAC-mediated bacteriolysis (Woodruff *et al.*, 2011 ▶). Various targeting approaches have already been explored, including monoclonal antibodies against C5a or C5aR (Sprong *et al.*, 2003 ▶; Lee *et al.*, 2006 ▶), small C5aR inhibitory molecules (Finch *et al.*, 1999 ▶), inhibitory peptides (Tokodai *et al.*, 2010 ▶), anti-C5a vaccines (Kessel *et al.*, 2014 ▶) and l-RNA aptamers (Hoehlig *et al.*, 2013 ▶). C5a-derived receptor antagonists have also been sought using phage-display libraries. This screening identified a mutant form of C5a, C5a-A8^Δ71–73^, as a potent antagonist of both C5aR and C5L2 (Otto *et al.*, 2004 ▶). This mutant lacks the three C-terminal residues of C5a-desArg (Δ^71^QLG^73^ in C5a numbering or Δ^748^QLG^750^ in C5 prepro numbering) and bears three additional mutations in its C-terminus (Fig. 1 ▶ *a*). In particular, the D746R mutation turned out to determine the antagonistic *versus* agonistic effect of these proteins (Otto *et al.*, 2004 ▶). However, the druggability of all these compounds still awaits final validation by clinical studies.

In light of the role played by C5a receptor signalling in inflammation, understanding the structure–function relationship of the interaction of C5a with C5aR/C5L2 appears to be a critical prerequisite for the design of more efficient therapeutics. Since mutations in the C-terminus and the removal of the C-terminal arginine modulate the activity of C5a towards its receptors, gaining insight into how these changes affect the three-dimensional architecture of the anaphylatoxin might provide clues to the mechanistics behind the C5aR antagonism of the truncated C5a proteins. C5a folds into a four-helix bundle in the structure of intact human C5 (Fredslund *et al.*, 2008 ▶) and in NMR structures of both human C5a (hC5a) and porcine C5a-desArg (Zuiderweg *et al.*, 1989 ▶; Williamson & Madison, 1990 ▶; Zhang *et al.*, 1997 ▶). Intriguingly, the crystal structure of human C5a-desArg (hC5a-desArg) revealed a distinct architecture for the protein, with a three-helix bundle core and the N-terminal helix either extruding outside the core or merging with the second helix into one single extended α-helix (Cook *et al.*, 2010 ▶). Although this property is not observed for C3a-desArg (Bajic *et al.*, 2013 ▶), it may still be of importance for the modulation of C5a activity. To investigate the conformational differences between the C5a-derived molecules, we have undertaken structural studies of C5a, C5a-desArg and C5a-A8^Δ71–73^ (referred to as C5a-A8 in the following). Since current protocols for C5a preparation rely either on isolation from plasma (Williamson & Madison, 1990 ▶), which yields heterogenous samples, or on harsh, time-consuming denaturation/renaturation procedures to purify recombinant proteins (Toth *et al.*, 1994 ▶; Cook *et al.*, 2010 ▶), we developed a simple and fast protocol to prepare biologically active C5a proteins by recombinant expression in bacteria for both human and mouse proteins. Furthermore, we determined the crystal structure of human C5a-A8 at 2.4 Å resolution. Interestingly, hC5a-A8 forms a three-helix bundle with an extended N-terminal helix as seen for hC5a-desArg. To assess whether this property was specific to the human C5a proteins or whether this is a general feature of C5a, we also determined the structures of mouse C5a and C5a-desArg (mC5a and mC5a-desArg) at 1.4 and 2.1 Å resolution, respectively. Both murine proteins fold into a four-helix bundle motif as observed for hC5a. Differences in the three-dimensional architecture of human and mouse C5a proteins are discussed in the light of their respective activity towards both human and mouse C5a receptors.

## 2. Materials and methods   

### 2.1. Genes and plasmids   

The hC5a gene, codon-optimized for bacterial expression and cloned between the *Bam*HI and *Eco*RI restriction sites of vector pET-32a (Novagen), was purchased from GenScript. To avoid nonspecific disulfide cross-linking between C5a molecules during purification, the sole cysteine residue of hC5a that is not engaged in intramolecular disulfide bridges (Cys704; prepro-C5 numbering) and is modified by cysteinylation in native hC5a (Laursen *et al.*, 2012 ▶) was replaced by an arginine, a substitution that is often encountered in C5a from other species (Supplementary Fig. S1^1^) and which does not dramatically affect the biological activity of C5a towards hC5aR (Hagemann *et al.*, 2006 ▶). To facilitate purification procedures, the enterokinase cleavage site preceding the hC5a sequence in pET-32a was replaced by a TEV protease cleavage site using the QuikChange Lightning site-directed mutagenesis kit from Agilent Technologies. Owing to difficulties with TEV protease cleavage in the initial purification trials, an additional four-amino-acid linker (AGAA) was introduced between the TEV cleavage site and the beginning of the hC5a sequence. hC5a-desArg and hC5a-A8 were generated from the hC5a-pET-32a construct by site-directed mutagenesis (using the QuikChange Lightning site-directed mutagenesis kit).

The mouse C5a (mC5a) gene, codon-optimized for bacterial expression, containing a 5′ insertion corresponding to a TEV protease cleavage site and cloned between the *Bam*HI and *Hin*dIII restriction sites of vector pET-32a (Novagen) was purchased from GenScript. The mC5a-desArg construct was derived from the mC5a-pET-32a construct by replacing the C-terminal Arg residue with a STOP codon (using the QuikChange Lightning site-directed mutagenesis kit). All constructs described here allow the expression of the protein of interest as a fusion with an N-terminal thioredoxin (Trx) tag followed by a hexahistidine tag and a TEV protease cleavage site (Trx-His_6_-TEV-C5a).

### 2.2. Expression and purification of C5a and derivatives   

All C5a constructs were transformed in Shuffle T7 Express *Escherichia coli* cells (New England Biolabs). The cells were grown at 37°C in 2×YT medium supplemented with ampicillin (100 µg ml^−1^) and expression of the C5a proteins was induced by the addition of 1 m*M* IPTG followed by overnight incubation at 18°C. The cells were harvested, resuspended in buffer *A* (50 m*M* HEPES pH 7.5, 300 m*M* NaCl, 30 m*M* imidazole, 1 m*M* PMSF) and disrupted by sonication. After clarification by centrifugation, the supernatant was applied onto a 5 ml Ni column (HisTrap FF column from GE Healthcare Life Sciences). After a high-salt wash (50 m*M* HEPES pH 7.5, 1 *M* NaCl, 30 m*M* imidazole, 1 m*M* PMSF) to remove nonspecifically bound proteins, the Trx-His_6_-tagged proteins were eluted with buffer *B* (50 m*M* HEPES pH 7.5, 300 m*M* NaCl, 500 m*M* imidazole, 1 m*M* PMSF). The Trx-His_6_ tag was subsequently removed by overnight incubation at 4°C using a 1:30 (rTEV:protein) mass ratio of in-house prepared recombinant His_6_-rTEV during dialysis against buffer *D* (50 m*M* HEPES pH 7.5, 300 m*M* NaCl, 0.5 m*M* EDTA). The cleaved proteins were separated from contaminants, His_6_-rTEV and uncleaved C5a material by a second Ni-column purification step. The protein samples were then concentrated and rediluted with 50 m*M* HEPES pH 7.5 to give a final salt concentration of 100 m*M* NaCl. The samples were then applied onto a 9 ml SOURCE 15S cation-exchange column (GE Healthcare Life Sciences). Elution was performed with a 100 ml linear gradient from 100 to 400 m*M* NaCl. Pure samples were flash-frozen in liquid nitrogen and kept at −80^o^C until use.

### 2.3. Crystallization of the C5a proteins   

Prior to crystallization, the buffer composition of the murine C5a proteins was adjusted to 20 m*M* HEPES pH 7.5, 150 m*M* NaCl and the protein samples were concentrated to 25 mg ml^−1^ for hC5a-A8 and to 8–10 mg ml^−1^ for mC5a and mC5a-desArg. Initial crystallization experiments were carried out in 96-well sitting-drop plates using a Mosquito robot (TTP Labtech) and commercial screens from Hampton Research and Molecular Dimensions. For hC5a-A8, crystals appeared after one month at 4°C over a reservoir consisting of 20%(*v*/*v*) 2-propanol, 20%(*w*/*v*) PEG 4000, 0.1 *M* sodium citrate pH 5.6 with a protein:reservoir solution ratio of 2:1. For data collection, the crystals were cryoprotected by soaking them in the reservoir solution followed by flash-cooling in liquid nitrogen. For mC5a and mC5a-desArg, rod-shaped crystals appeared after a few days at 4°C over a reservoir consisting of 0.1 *M* sodium acetate pH 4.6, 2.0 *M* sodium formate. Crystal optimization, in particular screening for additives using Additive Screen from Hampton Research, was performed in hanging drops and allowed us to obtain larger single crystals. For mC5a, the best crystals were grown at 4°C in 0.1 *M* sodium tricitrate pH 3.7, 2.6 *M* sodium formate, 0.5%(*w*/*v*) polyvinylpyrrolidone K15. For mC5a-desArg, the best crystals appeared over a reservoir consisting of 0.1 *M* sodium acetate pH 4.3, 2.4 *M* sodium formate, 3%(*w*/*v*) d-glucose monohydrate. For data collection, the crystals were cryoprotected by soaking them in a reservoir solution supplemented with either 4.0 *M* sodium formate (mC5a) or 30% glycerol (mC5a-desArg) followed by flash-cooling in liquid nitrogen.

### 2.4. Data collection and structure determination   

Data sets for hC5a-A8 were collected at 100 K on beamline X06SA at the Swiss Light Source (PSI, Villigen, Switzerland). Data sets for mC5a and mC5a-desArg were collected at 100 K on beamline 911-3 at MAX-lab (Lund, Sweden). All data sets were processed with *XDS* (Kabsch, 1993 ▶; Table 1 ▶). The hC5a-A8 crystals displayed *C*222_1_ symmetry and diffracted to a maximal resolution of 2.4 Å. The structure was solved by molecular replacement (MR) in *Phaser* (McCoy *et al.*, 2005 ▶) using a search model derived from the hC5a-desArg structure (Cook *et al.*, 2010 ▶) and encompassing residues Val694–Ser743 (*i.e.* where the N-terminal helix had been removed). Initial electron-density maps showed clear additional density for the missing residues in the hC5a-A8 N-terminus, which were rebuilt using *phenix.autobuild* (Adams *et al.*, 2010 ▶). The model was further improved by manual rebuilding in *Coot* (Emsley *et al.*, 2010 ▶) and energy minimization in *phenix.refine* (Adams *et al.*, 2010 ▶) using individual isotropic ADPs, TLS refinement and NCS restraints. The final model yielded *R*_work_ and *R*_free_ values of 22.4 and 23.8%, respectively, and its quality was evaluated with *MolProbity* (Chen *et al.*, 2010 ▶). The small gap observed between the *R*_work_ and *R*_free_ values for this model may be owing to the presence of the fourfold NCS, possibly leading to correlation between reflections in the test and the work sets, but is also favoured by the high overall data quality (Table 1 ▶) and measures taken throughout refinement to prevent overfitting. The mC5a and mC5a-desArg crystals both displayed *P*4_3_ symmetry with very similar unit-cell parameters and diffracted to 1.4 and 2.1 Å resolution, respectively (Table 1 ▶). The mC5a structure was solved by MR in *Phaser* using a homology model of mC5a based on the structure of hC5a extracted from the human C5 structure (Fredslund *et al.*, 2008 ▶). Initial electron-density maps obtained after MR using an mC5a model where the first 20 residues had been deleted (*i.e.* starting at residue Pro699 and encompassing only three helices) revealed clear additional density for the 20 missing residues in all four molecules of the asymmetric unit and showed that the mC5a N-terminus formed a fourth helical motif that packed against the remaining three helices in a four-helix bundle motif. Further refinement of the model was carried out by alternating between cycles of manual rebuilding in *Coot* and cycles of energy minimization with *phenix.refine* using individual ADPs with anisotropic ADP refinement for protein atoms and isotropic ADP refinement for water and ligand atoms. NCS restraints were not imposed as they were observed to increase the *R*_work_ and *R*_free_ values during refinement. H atoms were included as riding atoms during refinement but were removed from the final model. The final model yielded *R*_work_ and *R*_free_ values of 14.4 and 17.4%, respectively. The mC5a-desArg structure was solved by MR in *Phaser* using the mC5a structure as a search model. Refinement of the model was then carried out by alternating cycles of manual rebuilding in *Coot* and cycles of energy minimization with *phenix.refine* using individual ADP (isotropic) refinement as well as TLS refinement. NCS restraints were used throughout except for the very last refinement cycles. The final model yielded *R*_work_ and *R*_free_ values of 18.0 and 22.4%, respectively. The quality of the models was assessed using *MolProbity*. All figures were produced with *PyMOL* v.0.99rc6 (http://www.pymol.org).

### 2.5. Mass-spectrometric analysis of the human and mouse C5a proteins   

Approximately 5 µg purified protein (∼5 nmol) was desalted using C8 StageTips (Proxeon) and the bound material was eluted with 90% acetonitrile, 0.08% trifluoroacetic acid. The material was lyophilized, resuspended in 2 µl 1% trifluoroacetic acid and mixed with 2 µl 2,5-dihydroxyaceto­phenone [0.1 *M* in 20 m*M* ammonium dihydrogen citrate, 75%(*v*/*v*) EtOH] (Wenzel *et al.*, 2006 ▶). The material (1 µl) was spotted onto a stainless-steel target and allowed to dry. The spectra were recorded in positive and linear mode using an AutoFlex Smartbeam III instrument (Bruker) calibrated by external calibration (Peptide Calibration Standard I, Bruker Daltronics). The centroid masses determined were evaluated using the *GPMAW* software (http://www.gpmaw.com).

### 2.6. *N*-Acetyl-β-d-glucosaminidase release assay (GARA)   

Prior to the GARA, bacterial endotoxin was removed from all recombinant C5a samples using a Detoxi-Gel Endotoxin Removing Column (Pierce). The release of the lysosomal enzyme *N*-acetyl-β-d-glucosaminidase was assayed as described previously (Gerard & Gerard, 1990 ▶; Bajic *et al.*, 2013 ▶). Briefly, rat basophil leukaemia (RBL) cells stably transfected with the retroviral vector pQCXIN encoding human C5aR, mouse C5aR or an empty cassette (as a negative control) were harvested and resuspended in HAG-CM-complete buffer (20 m*M* HEPES pH 7.4, 125 m*M* NaCl, 5 m*M* KCl, 1 m*M* CaCl_2_, 1 m*M* MgCl_2_, 0.5 m*M* glucose, 0.25% BSA) at a concentration of 2 × 10^6^ cells ml^−1^ and incubated at 37°C for 20 min. Simultaneously, dilutions of anaphylatoxins were incubated with 1 m*M* cytochalasin B at 37°C. 75 µl of the cell suspension was added to each C5a dilution and release was allowed to proceed for 3 min at 37°C. The reactions were then transferred to ice and centrifuged for 3 min at 400*g* and 4°C. NAG activity was assessed in the presence of 3.53 m*M* *p*-nitrophenyl-*N*-acetyl-β-d-glucosamide (Sigma) for 60 min at 37°C. The enzymatic reaction was stopped by adding 0.4 *M* glycine–NaOH pH 10.4 and the spectral absorbance was measured at 405 nm. The maximal NAG release (100% reference) corresponds to the maximal NAG release activity obtained for commercial hC5a. The data are the means ± standard deviation of multiple independent experiments and were analyzed in *GraphPad Prism* (http://http://www.graphpad.com) using a four-parameter logistic nonlinear regression. Statistical analyses were performed using the paired two-tailed Student’s t-test in *Excel*.

## 3. Results   

### 3.1. Production of biologically active anaphylatoxins   

Human C5a, C5a-desArg and C5a-A8 (Fig. 1 ▶ *a*) were expressed as recombinant proteins in *E. coli* and purified using the same procedure as previously described for human C3a and C3a-desArg (Bajic *et al.*, 2013 ▶). Since the C5a molecule is stabilized by three internal disulfide bridges, we used an *E. coli* strain enhancing disulfide-bond formation and expressed the proteins as fusions with a cleavable thioredoxin tag to ensure proper folding. The final step of the purification consisted of cation-exchange chromatography. All three proteins eluted as a single monodisperse peak on the SOURCE 15S column (Fig. 1 ▶ *b*) at NaCl concentrations correlating with their isoelectric properties (205, 190 and 230 m*M* NaCl for hC5a, hC5a-desArg and hC5a-A8, respectively), and 20–25 mg of purified protein per litre of bacterial culture was routinely obtained using this method. Mass-spectrometric analysis of all three purified samples also gave molecular masses in agreement with theoretical values (Supplementary Fig. S2).

To verify that the procedure yielded biologically active anaphylatoxins, the recombinant proteins were tested in the *N*-acetyl-β-d-glucosaminidase release assay (GARA) using RBL cells stably transfected with human C5aR (Gerard & Gerard, 1990 ▶; Bajic *et al.*, 2013 ▶; Figs. 1 ▶ *c* and 1 ▶ *d*). Recombinant hC5a triggered *N*-acetyl-β-d-glucosaminidase (NAG) release in a dose-dependent manner, with an EC_50_ value of 1.31 ± 0.47 n*M*, comparable to the value of 1.47 ± 0.43 n*M* obtained with commercially available recombinant hC5a (Altana Pharma AG). hC5a-desArg could also activate hC5aR-expressing cells with an EC_50_ value of 1.44 ± 0.98 n*M*, similar to the value obtained with hC5a. However, the maximal NAG release activity observed for hC5a-desArg only reached 55% of the hC5a-induced maximal activity (Figs. 1 ▶ *c* and 1 ▶ *d*). Thus, our recombinant hC5a-desArg acts as a partial agonist of hC5aR, in good agreement with previous reports (Bürgi *et al.*, 1994 ▶; Higginbottom *et al.*, 2005 ▶). Finally, no activation could be detected upon incubation with hC5a-A8, even at concentrations of up to 1 µ*M* (Figs. 1 ▶ *c* and 1 ▶ *d*): a behaviour in agreement with the antagonistic properties previously described for hC5a-A8 (Otto *et al.*, 2004 ▶). In conclusion, the purification protocol described here yielded highly homogenous, fully functional recombinant protein suitable for crystallographic studies.

### 3.2. Human C5a-A8 folds into a three-helix bundle with a C-terminal β-strand extension   

#### 3.2.1. Overall structure of hC5a-A8   

Since the crystal structure of hC5a-desArg was already available and, surprisingly, revealed a three-helix bundle conformation (Cook *et al.*, 2010 ▶), we decided to further investigate the structural differences that possibly exist between the C5a-derived proteins. No crystal structure of recombinant hC5a has been reported so far and, despite numerous attempts, we did not obtain hC5a crystals, in part owing to the very high solubility of the protein in most crystallization conditions. On the other hand, hC5a-A8 produced crystals displaying *C*222_1_ symmetry and diffracting to 2.4 Å resolution (Table 1 ▶). Initial attempts to solve the structure using the C5a moiety (residues 679–742) from the hC5 structure (Fredslund *et al.*, 2008 ▶) failed to give a molecular-replacement (MR) solution. Since the conserved core between all reported hC5a structures consists of a three-helix bundle encompassing helices H2, H3 and H4 (Zuiderweg *et al.*, 1989 ▶; Zhang *et al.*, 1997 ▶; Fredslund *et al.*, 2008 ▶; Cook *et al.*, 2010 ▶), a model based on the hC5a-desArg structure and lacking the N-terminal helical segment (*i.e.* residues Val694–Ser743 of monomer *A* from PDB entry 3hqa; Cook *et al.*, 2010 ▶) was then used in the MR search. This three-helix bundle model gave a clear MR solution (log-likelihood gain of 946) and initial electron-density maps displayed well defined additional density for the missing N-terminal residues (Supplementary Fig. S3*a*). The final model encompasses residues Thr678–Lys745 (residues 1–68 in C5a numbering) and the penultimate hC5a-A8 residue, Arg746, could be modelled in one of the four molecules related by NCS in the asymmetric unit (monomer *C*). The four NCS-related molecules are very similar overall, with average r.m.s.d. values of 0.75 and 1.35 Å on C^α^ atoms and all atoms, respectively, between all four NCS-related molecules (residues Thr678–Lys745). Only one molecule (monomer *A*) was therefore later used for comparison with other C5a proteins.

**Figure 2:**
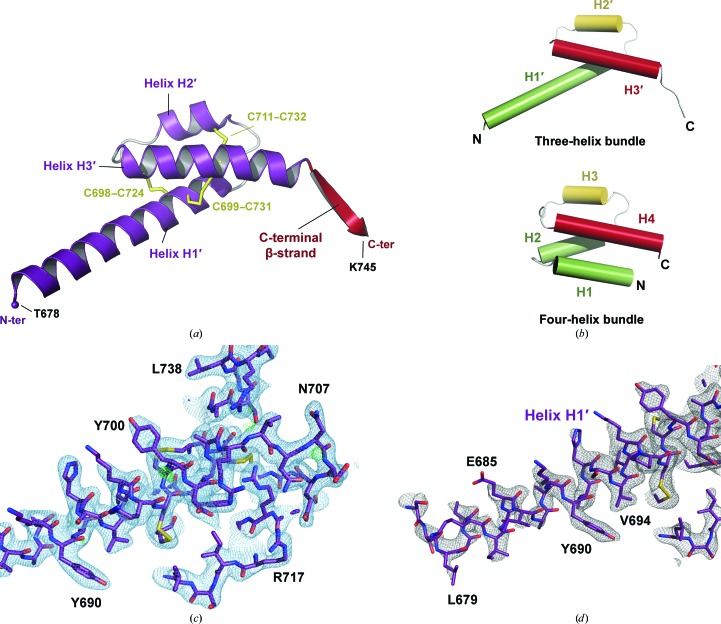
General overview of the hC5a-A8 structure. (*a*) Overall structure of human C5a-A8 at 2.4 Å resolution. (*b*) Comparison of the three-helix bundle of hC5a-A8 with the four-helix bundle of hC5a as part of C5 (Fredslund *et al.*, 2008 ▶). (*c*) Final model and electron-density maps for the hC5a-A8 structure. The 2*mF*_o_ − *DF*_c_ map is shown as a blue mesh and contoured at 1σ. The *mF*_o_ − *DF*_c_ map is shown as a green mesh and contoured at 3σ. (*d*) OMIT electron-density map calculated using simulated annealing from a model where residues Thr678–Ser693 (the first half of helix H1′) have been deleted. The 2*mF*_o_ − *DF*_c_ OMIT map is shown as a grey mesh and contoured at 1σ. The final complete model is superimposed to show the quality of the fit.

The overall structural arrangement of hC5a-A8 is depicted in Fig. 2 ▶. The C5aR antagonist does not fold into a four-helix bundle but instead adopts a three-helix bundle conformation with a long N-terminal helix H1′ encompassing residues Thr678–Arg704 (Cys704 in native hC5a), a second helix H2′ formed by residues Thr710–Ile718 and the last helix H3′ comprising residues Gly721–Asn741 (Fig. 2 ▶ *a*). Helices H2′ and H3′ from hC5a-A8 correspond to helices H3 and H4, respectively, in the canonical four-helix bundle conformation of hC5a (Zhang *et al.*, 1997 ▶; Fredslund *et al.*, 2008 ▶), while the first two helices in hC5a, H1 and H2, are now merged into a single extended helix H1′ in hC5a-A8 (Fig. 2 ▶ *b*, Supplementary Fig. S1). Despite these major differences, the three disulfide bridges that stabilize the helix packing in all previously reported C5a structures are preserved in hC5a-A8 and connect Cys698–Cys724 (H1′–H3′), Cys699–Cys731 (H1′–H3′) and Cys711–Cys732 (H2′–H3′).

#### 3.2.2. Comparison with other C5a structures   

**Figure 3:**
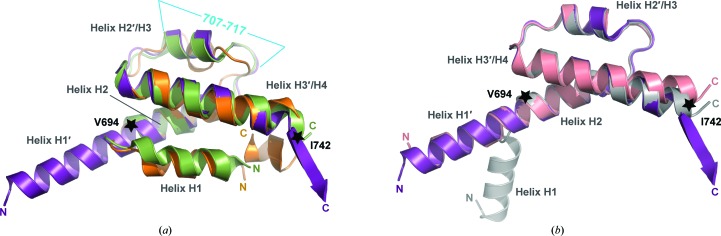
Comparison of hC5a-A8 with other human C5a structures. In both panels, the residues delimitating the conserved core between all structures, Val694 and Ile742, are indicated with stars. (*a*) Superimposition of hC5a-A8 (purple) with the structure of hC5a from human C5 (green; PDB entry 3cu7; Fredslund *et al.*, 2008 ▶) and from the NMR model (orange; PDB entry 1kjs; Zhang *et al.*, 1997 ▶). The stretch of residues corresponding to the region encompassing the most significant structural differences within the conserved core of all three proteins is indicated in blue. (*b*) Superimposition of hC5a-A8 (purple) with the two conformations observed for hC5a-desArg (grey and salmon for monomers *A* and *B*, respectively, from PDB entry 3hqa; Cook *et al.*, 2010 ▶).

The core of hC5a-A8 encompassing the second half of helix H1′, helix H2′ and helix H3′ (residues Val694–Ile742) is well conserved compared with all reported hC5a/hC5a-desArg structures (Fig. 3 ▶). The r.m.s.d. on C^α^ atoms for this region in hC5a-A8 is 0.99 Å compared with hC5a from C5 (Fredslund *et al.*, 2008 ▶) and 0.55 and 0.90 Å compared with conformers *A* (the conformer with four helices) and *B* (the conformer with three helices) of hC5a-desArg, respectively (Cook *et al.*, 2010 ▶). A higher r.m.s.d. value of 2.46 Å is encountered upon comparison with the mean structure of the hC5a NMR structure (Zhang *et al.*, 1997 ▶) owing to a distortion of the region between Val705 and Ala716 in the NMR model compared with other C5a structures (Fig. 3 ▶ *a*).

In hC5a, the short sequence preceeding the core region (residues Tyr690–Ser693) forms a loop that allows the N-terminal segment to pack back against the C5a core in an H1 helical motif antiparallel to H2. In contrast, residues Tyr690–Ser693 of hC5a-A8 form an additional helical turn prior to H2, allowing the latter to merge with the N-terminal helical segment into the long helix H1′. This arrangement is reminiscent of that observed for hC5a-desArg (Cook *et al.*, 2010 ▶). Indeed, monomer *B* of hC5a-desArg (PDB entry 3hqa) displays a similar structural arrangement to hC5a-A8, with a long N-terminal helix and an overall three-helix bundle architecture, and the two structures superimpose well with an average r.m.s.d. on C^α^ atoms of 1.45 Å for the region encompassing residues Lys682–Arg739 (Fig. 3 ▶ *b*).

Packing in the hC5a-A8 crystals is primarily stabilized by two major interactions occurring for each of the four monomers in the asymmetric unit (Supplementary Fig. S3*b*). The first interaction allows the long helix H1′ to pack along its counterpart from a symmetry-related molecule in an antiparallel manner. The second interaction bridges the C-termini of two symmetry-related molecules in an antiparallel β-sheet (see below). In addition, the first residues of the H1′ N-terminus pack against the H1′–H2′ loop of another molecule. Finally, hydrogen bonds between adjacent monomers further stabilize the packing. A crystal-packing dimer present in the asymmetric unit of the hC5a-desArg crystals led (Cook *et al.*, 2010 ▶) to the proposal that formation of such a C5a dimer might be relevant *in vivo*, for example in promoting C5aR oligomerization, and that the switch to the C5a-desArg conformation compared with the four-helix bundle could allow regulation of C5a activity by blocking access to some of the residues involved in C5aR binding. The packing in the hC5a-A8 crystals differs quite extensively from the packing in the hC5a-desArg crystals. Analysis with *PISA* (Krissinel & Henrick, 2007 ▶) reveals that the strongest dimeric interface is formed by the H1′–H1′ antiparallel assembly, with a total buried surface area of 1500 Å^2^, followed by the packing of two hC5a-A8 molecules along the concave side of their cores formed by helices H1′ and H3′ (total buried surface area of 1180 Å^2^). The other monomer–monomer interactions that are observed in the hC5a-A8 crystals do not represent biologically relevant interfaces according to *PISA*. In any case, none of these interactions match those observed in the hC5a-desArg structure. Taken together, these data suggest that the dimeric assemblies observed in both structures are solely promoted by crystal packing and are most probably not relevant for the biological function of the proteins.

#### 3.2.3. A new conformation of the C5a C-terminus   

**Figure 4:**
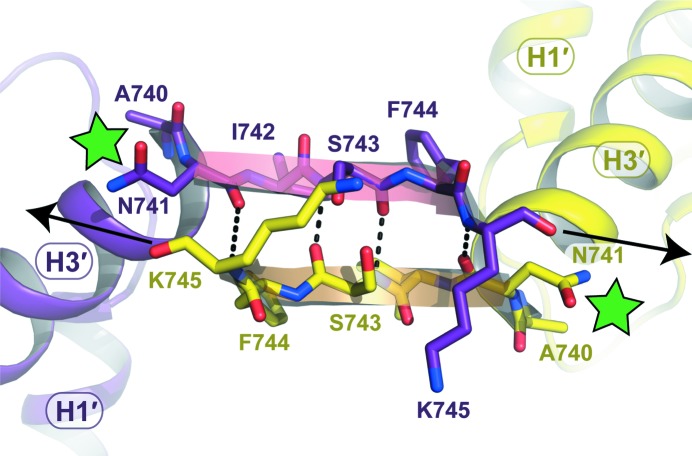
The hC5a-A8 C-terminal β-strand extension. Close-up view of the hC5a-­A8 C-terminal region adopting a β-strand structure which extends outside the three-helix bundle core. The formation of a two-stranded antiparallel β-sheet between the C-termini of two hC5a-A8 molecules participates in the packing within hC5a-A8 crystals. The residues involved in stabilizing interactions between the two strands are shown as sticks. The position of the carbohydrate chain protruding from Asn741 in native, glycosylated hC5a is indicated by green stars. Black arrows indicate the direction in which the peptide chain could extend in full-length hC5a.

The C5a C-terminus (the residues following Ala740) is generally disordered and/or adopts multiple conformations in most C5a structures, reflecting the flexibility of this region. To date, it has only been traced in the NMR structure of hC5a, which provides an atomic model for all 74 C5a residues (Zhang *et al.*, 1997 ▶). In this NMR model, the hC5a C-terminus folds into a short α-helix encompassing two turns and packs back against the four-helix bundle in between helices H1 and H4. Although the C-terminus of hC5a-A8 is shortened compared with hC5a, it could be traced almost entirely for all four molecules in the asymmetric unit. Interestingly, it does not form a helical motif but is instead arranged in a β-strand linking Asn741 to Lys745 that extends outside the C5a core, prolonging helix H3′ (Figs. 2 ▶ *a* and 4 ▶). This short β-strand interacts with the same region from a symmetry-related molecule to form an antiparallel two-stranded β-sheet (Fig. 4 ▶). This β-sheet is maintained by four hydrogen bonds between main-chain atoms from each monomer: Asn741 O–Lys745 N, Ser743 O–Ser743 N and the symmetrical interactions. The residues contained in this β-strand are all conserved in full-length hC5a, except for the His744Phe substitution. This β-strand structure may therefore not be restricted to hC5a-A8 and could also be adopted by the hC5a/hC5a-desArg C-terminus, for example upon binding to C5aR or C5L2. However, native hC5a and hC5a-desArg are glycosylated on Asn741, as seen in the structure of hC5 (Fredslund *et al.*, 2008 ▶). Carbohydrate moieties protruding from the Asn741 side chain might therefore affect the ability of the C-terminal region of hC5a to dimerize *via* β-sheet formation (Fig. 4 ▶). Thus, whether the association of the C5a C-terminus in a β-sheet with another C5a molecule would occur *in vivo* or only reflects a packing artefact has yet to be determined. Although the *in vivo* concentrations of C5a and C5a-desArg are generally considered to be rather low, several mechanisms have now been reported that can produce complement anaphylatoxins independently of complement activation, both extracellulary and intracellularly (Huber-Lang *et al.*, 2006 ▶; Amara *et al.*, 2010 ▶; Liszewski *et al.*, 2013 ▶), thus locally generating a high anaphylatoxin concentration which could be favourable for anaphylatoxin dimerization. Such a β-sheet pairing between C5a molecules could therefore occur in specific cellular contexts.

### 3.3. Mouse C5a and C5a-desArg adopt the canonical four-helix bundle conformation   

**Figure 5:**
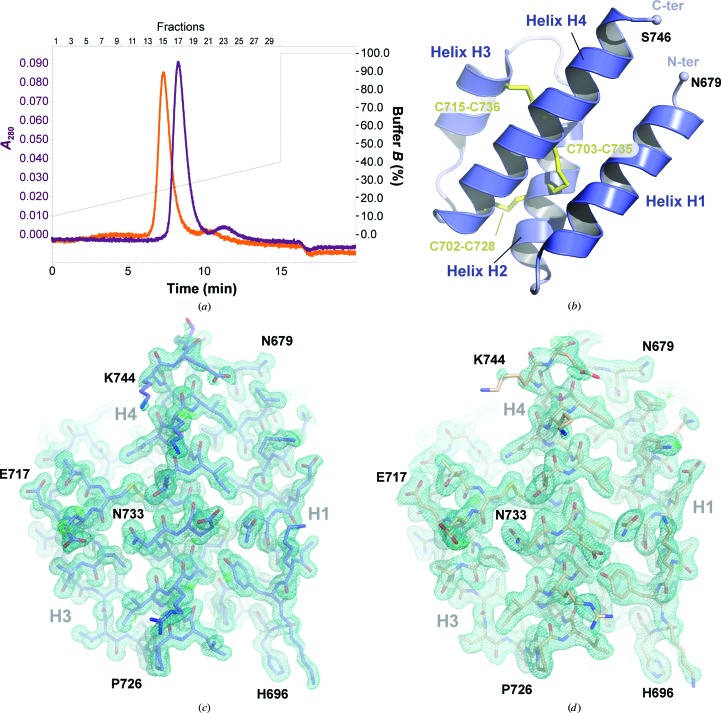
General overview of mC5a and mC5a-desArg structures. (*a*) Elution profile of recombinant mC5a (purple) and mC5a-desArg (orange) on the SOURCE 15S column. (*b*) Overall structure of mouse C5a at 1.4 Å resolution. The four α-helices are labelled according to the hC5a nomenclature and the three internal disulfide bridges stabilizing the four-helix bundle are highlighted in yellow. (*c*) Final model and electron-density maps for the mC5a structure. The 2*mF*_o_ − *DF*_c_ map is shown as a blue mesh and contoured at 1σ. The *mF*_o_ − *DF*_c_ map is show in blue/green and contoured at 3σ. (*d*) As in (*c*) but for the mC5a-desArg structure.

The three-helix bundle conformation has only been observed so far for hC5a-derived molecules, since porcine C5a-desArg folds into a four-helix bundle (Williamson & Madison, 1990 ▶). We therefore wanted to investigate whether this was a specific property of the human proteins and/or whether this was dependent on the truncation of the C5a C-terminus. For this purpose, we undertook structural studies of the mouse C5a and C5a-desArg proteins which share 63.5% sequence identity with hC5a (Supplementary Fig. S1). mC5a and mC5a-desArg were prepared recombinantly using the same protocol as used for the human proteins. The procedure again yielded very homogenous samples as judged by their elution profile on the SOURCE 15S column (Fig. 5 ▶ *a*) and their analysis by mass spectrometry (Supplementary Fig. S2). However, the overall yield was much lower than for the human proteins, with an average of 200–500 µg purified protein per litre of bacterial culture.

mC5a and mC5a-desArg crystals both displayed *P*4_3_ symmetry with very similar unit-cell parameters and diffracted to 1.4 and 2.1 Å resolution, respectively (Table 1 ▶). Initial attempts to solve the mC5a structure by MR were made using the four-helix bundle model of hC5a (Fredslund *et al.*, 2008 ▶). This gave a clear solution, and final refinement of the model to *R*_work_ and *R*_free_ values of 14.42 and 17.40%, respectively, confirmed the mC5a structural arrangement (Table 1 ▶). The mC5a model was then used to solve the structure of mC5a-desArg. Again, a clear MR solution could be obtained with the four-helix bundle model. For both mC5a and mC5a-desArg, an MR search performed using the same three-helix bundle model as for hC5a-A8 produced initial electron-density maps that displayed a clear additional density for the missing N-terminal residues (Supplementary Fig. S4), in agreement with a canonical four-helix bundle motif. The final mC5a-desArg model was refined to *R*_work_ and *R*_free_ values of 18.03 and 22.41%, respectively (Table 1 ▶).

**Figure 6:**
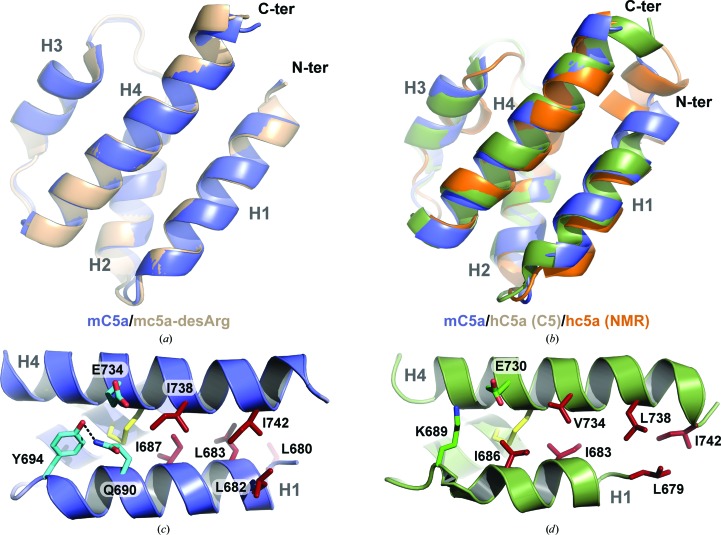
Comparison of the mC5a/mC5a-desArg structures with hC5a. (*a*) Superimposition of the mC5a (blue) and mC5a-desArg (beige) structures. (*b*) Superimposition of mC5a (blue) with the structure of hC5a from human C5 (green; Fredslund *et al.*, 2008 ▶) or from the NMR model (orange; Zhang *et al.*, 1997 ▶). (*c*, *d*) Stabilizing interactions between helices H1 and H4 in the four-helix bundle models of mC5a (blue) and hC5a (green). The residues involved in hydrogen bonds, salt bridges and hydrophobic interactions at the H1–H4 interface are represented as sticks in both structures.

The structure of mC5a is depicted in Fig. 5 ▶(*b*). As the mC5a-desArg structure is very similar to the mC5a structure (see below), the mC5a-desArg model was not displayed in Fig. 5 ▶. In both structures, the atomic model was traced between residues Asn679 and Ser746, which corresponds to the four-helix bundle core, with helix H1 extending between Asn679 and Tyr694, helix H2 formed by residues Ser697–Arg708, helix H3 encompassing residues Thr714–Val722 and helix H4 comprising residues Gly725–Ser746. The overall fold is also stabilized by three disulfide bridges in both mC5a and mC5a-desArg: Cys702–Cys728 (H2–H4), Cys703–Cys735 (H2–H4) and Cys715–Cys736 (H3–H4). Superimposition of mC5a and mC5a-desArg reveals a high similarity between the two structures, with an average r.m.s.d. on C^α^ atoms of 0.36 Å (Fig. 6 ▶ *a*), and corresponding residue side chains adopt similar positions in the two structures. mC5a and mC5a-desArg also superimpose well with hC5a (Fig. 6 ▶ *b*), with r.m.s.d.s on C^α^ atoms of 1.24 and 1.23 Å, respectively, compared with the C5a moiety from human C5 (Fredslund *et al.*, 2008 ▶). Small differences are mostly encountered for the loop regions connecting the different helices. Thus, the canonical four-helix bundle fold is well conserved between species.

The C-terminus of mC5a and mC5a-desArg (region Pro747–Arg755/Gly754) was not visible in the electron density, again suggesting its high flexibility. Surprisingly, a blob of density that assumed the shape of an arginine was visible in the mC5a structure (Supplementary Fig. S5). This density is observed in the groove between the helix H1 N-terminus and the helix H2 C-terminus of monomer *A* in the asymmetric unit and is located within hydrogen-bonding distance of Arg743 of the same monomer (Supplementary Fig. S5*a*). The density is less prominent around this position in the other monomers of the mC5a asymmetric unit, but this density is not observed around any of the molecules present in the mC5a-desArg crystals, although mC5a-desArg crystallizes under the same conditions and according to the same packing as mC5a (Supplementary Fig. S5*b*). Furthermore, none of the components present either in the protein buffers or in the crystallization conditions could account for such a density. Although it cannot be ruled out that this corresponds to a ligand trapped by the protein during bacterial expression, modelling of an arginine residue at this position matches the density perfectly (Supplementary Fig. S5*c*). Inspection of the crystal packing reveals that this density could represent the C-terminal Arg755 of mC5a from a neighbouring symmetry-related molecule, in which the C^α^ atom of the last modelled residue, Glu745, is separated by 21 Å from the putative position of this Arg755 C^α^ atom (Supplementary Fig. S5*d*), a distance that could accommodate a stretch of nine residues assuming a random-coil nonlinear conformation. Thus, the position of Arg755 could have been stabilized by packing against Arg743 from a symmetry-related molecule, while the connecting residues Ser746–Gly754 adopt multiple conformations within the solvent separating the two layers of molecules. As we cannot attribute this density to Arg755 without ambiguity, the density was left unmodelled in the final mC5a structure.

### 3.4. Differential activity of C5a/C5a-desArg anaphylatoxins across species   

**Figure 7:**
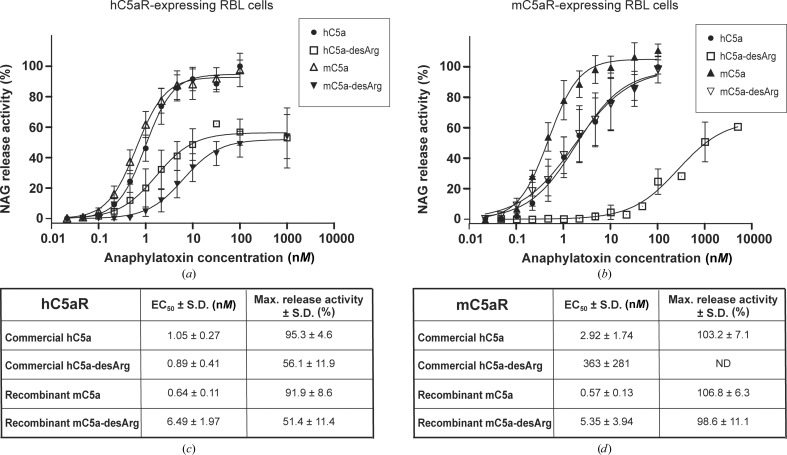
GARA for recombinant mC5a and mC5a-desArg against both hC5aR and mC5aR and comparison with commercial hC5a/hC5a-desArg. All results are indicated as the mean ± standard deviation of at least eight individual experiments (except for hC5a-desArg, where *n* = 4). The NAG release activity is expressed as a percentage of the maximal NAG release activity obtained with commercial hC5a. The statistical significance of the differential behaviour observed between hC5a and mC5a towards both receptors is as follows: *p*-value (hC5aR) = 0.0016; *p*-value (mC5aR) = 0.006. (*a*) Activation of hC5aR-expressing RBL cells by recombinant mC5a and mC5a-desArg compared with commercially available recombinant hC5a and hC5a-desArg. (*b*) As in (*a*) but with mC5aR-expressing RBL cells. (*c*) EC_50_ and maximal NAG release activity observed for all proteins upon incubation with hC5aR-expressing RBL cells. (*d*) As in (*c*) but with mC5aR-expressing RBL cells.

In the light of the structural differences observed between human and mouse C5a-desArg, we decided to evaluate whether these differences could reflect their respective activation properties towards both human and murine C5a receptors. For this purpose, mC5a and mC5a-desArg were first tested in the GARA against RBL cells expressing human C5aR. As shown in Figs. 7 ▶(*a*) and 7 ▶(*c*), recombinant mC5a can activate hC5aR in a dose-dependent manner to the same level as commercially available recombinant hC5a and with an even lower EC_50_ (0.64 ± 0.11 n*M* on average compared with 1.05 ± 0.27 n*M* for commercial hC5a). mC5a-desArg could also activate hC5aR but to a maximal NAG release activity that reached only half of the activity observed for mC5a and hC5a. Thus, as hC5a-desArg, mC5a-desArg also acts as a partial agonist of hC5aR. However, while hC5a-desArg retains an EC_50_ equivalent to that measured for hC5a, mC5a-desArg has a tenfold higher EC_50_ than mC5a.

All four anaphylatoxins were then tested against the murine C5a receptor in the GARA (Figs. 7 ▶ *b* and 7 ▶ *d*). As expected, mC5a is a full agonist of mC5aR and can activate the receptor with an EC_50_ of 0.57 ± 0.13 n*M*. Surprisingly, mC5a-desArg could activate mC5aR to the same level as mC5a, although with a much increased EC_50_ of 5.35 ± 3.94 n*M*. Thus, in contrast to the activity ofC5a-desArg towards hC5aR, mC5a-desArg seems to act as a full agonist of mC5aR but with a lower potency. For comparison, the biological activity of commercially available recombinant hC5a and hC5a-desArg towards mC5aR was then assessed. Intriguingly, while mC5a and mC5a-desArg can substitute for their human counterparts with respect to hC5aR activation, the reverse does not hold true. Indeed, hC5a could activate mC5aR to the same extent as mC5a but with a fivefold higher EC_50_, thereby acting more similarly to mC5a-desArg. In contrast, the maximal NAG release observed upon incubation with up to 10 µ*M* hC5a-desArg reached only 60% of the maximal activity obtained with any of the three other anaphylatoxins, with an EC_50_ that was on average 100 times higher than for hC5a or mC5a-desArg. Thus, hC5a-desArg only acts as a partial agonist towards mC5aR, thereby failing to match the behaviour of mC5a-desArg with respect to mC5aR.

Taken together, these data show that hC5a and mC5a both act as C5aR agonists independently of the receptor species and that in both cases mC5a is a more potent agonist than hC5a. In contrast, the desArg versions of these anaphylatoxins have differential effects depending on the receptor species. Indeed, hC5a-desArg is a partial agonist of both hC5aR and mC5aR but has a much reduced potency towards mC5aR, whereas mC5a-desArg only acts as a partial agonist towards hC5aR and is a full agonist towards mC5aR but with a reduced potency compared with mC5a.

## 4. Discussion   

Here, we have reported the crystal structures of the human C5aR antagonist hC5a-A8 and of the two murine anaphylatoxins mC5a and mC5a-desArg. The proteins were produced recombinantly in bacteria using a fast and simple protocol previously employed to purify recombinant bioactive hC3a and hC3a-desArg (Bajic *et al.*, 2013 ▶). As judged for the human proteins, this procedure yielded recombinant proteins possessing the same biological activity towards hC5aR as commercially available C5a proteins, with potencies and agonist/antagonist effects that are in agreement with the current literature (Otto *et al.*, 2004 ▶; Higginbottom *et al.*, 2005 ▶). Interestingly, the hC5a-A8 antagonist crystallized as a three-helix bundle similar to that recently described for hC5a-desArg (Cook *et al.*, 2010 ▶), whereas both mC5a and mC5a-desArg adopted the canonical four-helix bundle conformation also observed for hC5a both as a part of C5 (Fredslund *et al.*, 2008 ▶) and as an isolated protein in solution (Zhang *et al.*, 1997 ▶). Furthermore, evaluation of the anaphylatoxic properties of both human and murine proteins in an *N*-acetyl-β-d-glucosaminidase release assay revealed strong differences between species, *i.e.* of human *versus* murine proteins towards both hC5aR and mC5aR, and for the same species but towards cognate *versus* noncognate receptors. Such differences between species have also been reported for the human and porcine systems (Bubeck *et al.*, 1994 ▶). Since C5a sequences are well conserved across species (Supplementary Fig. S1), modulation of their activity towards C5a receptors could arise from subtle differences in both anaphylatoxins and receptors, in addition to different downstream effectors of C5aR activation. Furthermore, since C5a is rapidly desarginated *in vivo* after release from its inert C5 precursor, our findings that hC5a-desArg and mC5a-desArg have differential activation properties towards their cognate receptors indicate that desargination acts as a partial control mechanism mainly in humans but not in mice. Therefore, the biological effects of C5a/C5a-desArg in this rodent (*e.g.* in animal models *versus* diseases) might be more prominent and/or less locally restricted to the site of complement activation owing to the higher remaining biological activity of the C5a-desArg form, which represents 99% of the circulating C5a form in the blood. This might also compensate for the relatively low levels of complement factors, including C5, reported in mice (Ong & Mattes, 1989 ▶). In consequence, a clearly visible differential effect of C5a/C5a-desArg in C5aR^−/−^ *versus* wild-type mice can be expected, thereby validating the use of mouse models to study C5a/C5a-desArg-dependent effects despite the low C5/C5a levels in mice.

Extensive mutational studies have mapped the C5aR binding site on C5a (Mollison *et al.*, 1989 ▶; Toth *et al.*, 1994 ▶; Bubeck *et al.*, 1994 ▶; Hagemann *et al.*, 2006 ▶, 2008 ▶). It is now commonly accepted that C5a binding to its cognate receptor occurs through a two-site binding mode (Siciliano *et al.*, 1994 ▶). The C5a core first docks against the receptor N-terminus, allowing proper positioning of the C5a molecule with respect to the GPCR transmembrane region. The C5a C-terminus can then insert into a binding pocket within the C5aR transmembrane domain, thereby triggering receptor activation. The C5a core residues interacting with the receptor N-terminus are located either in the H1–H2 loop and at the beginning of helix H2 (four-helix bundle nomenclature) or in the region encompassing the end of helix H3, the H3–H4 loop and the beginning of helix H4 (Supplementary Fig. S1), *i.e.* on the opposite face of C5a compared with the C-terminal extension. This patch is mostly composed of positively charged residues, such as His692, Lys696, Lys697, Arg714, Arg717, Arg723 and Lys726, that interact with acidic residues and sulfotyrosines contained in the C5aR N-terminus (DeMartino *et al.*, 1994 ▶; Mery & Boulay, 1994 ▶; Chen *et al.*, 1998 ▶; Farzan *et al.*, 2001 ▶). Interestingly, none of these C5a residues are located within helix H1. This could indicate that C5a binding can occur independently of whether the anaphylatoxin adopts a three-helix or four-helix bundle conformation. In agreement with this idea, hC5a-desArg and mC5a-desArg display similar activation properties towards hC5aR, although their respective crystal structures (Cook *et al.*, 2010 ▶ and this study) reveal quite distinct structural arrangements of the two proteins. Evidently, one cannot rule out that the conformations of the molecules in the crystal structures differ from the conformations that they adopt upon receptor binding. Thus, although mC5a and mC5a-desArg adopt a four-helix bundle fold in the crystal structure, their conformation could change to a three-helix bundle upon receptor binding. Another possibility, as suggested previously (Cook *et al.*, 2010 ▶; Bajic *et al.*, 2013 ▶), is that the three-helix bundle conformation may be relevant for other C5a functions such as the processing of pro-C5 into separate α and β chains or the cleavage of C5a by carboxy­peptidases. Comparison of the H1–H4 packing in both hC5a and mC5a (Figs. 6 ▶ *c* and 6 ▶ *d*) reveals that the stabilization of the H1–H4 interface in mC5a is strengthened by the presence of an additional helical turn in the H1 N-terminus owing to the N-terminal extension by three residues (Asn-Leu-His) of mC5a compared with hC5a. However, this property may only be specific to mouse and rat C5a, since C5a proteins from other species have a length similar to hC5a in their N-termini (Supplementary Fig. S1). A similar situation was encountered for the hC3a and hC3a-desArg proteins (Bajic *et al.*, 2013 ▶), which both display a four-helix bundle conformation while acting as expected towards hC3aR (Wilken *et al.*, 1999 ▶). In the case of hC3a and hC3a-desArg, the H1–H4 packing again seemed to be more strongly stabilized than for hC5a, in which H1–H4 interactions are exclusively hydrophobic. In particular, hydrogen bonds between Lys682 (H1), Tyr686 (H1) and Asp726 (H2) further held helices H1 and H2 in place in the hC3a and hC3a-desArg structures. A similar triad is encountered in mC5a and mC5a-desArg with Gln690 (H1), Tyr694 (H1) and Glu734, although the Glu734 side chain points away from the residues in H1 and interacts with a symmetry-related molecule. Thus, the propensity of helix H1 from hC5a to separate from the four-helix bundle might be an intrinsic property of the molecule allowing this to occur more spontaneously, while a specific context (binding partner or biological function) may be required for mC5a-derived and/or hC3a-derived molecules.

In any case, the *in vivo* relevance of the three-helix bundle conformation with respect to C5aR activation and C5L2 binding remains to be established. Our study also revealed that anaphylatoxin properties are not equivalent among species. Thus, conformational modulation of the anaphylatoxin three-dimensional architecture may have differential effects depending on the receptor considered, as examplified by mC5a-desArg and hC5a-desArg, which behave similarly towards hC5aR but have quite distinct activities towards mC5aR. Such differential effects across species are quite intriguing, notably since both the C5a and C5aR sequences are quite well conserved (Supplementary Figs. S1 and S6). In particular, both the sulfotyrosines and the aspartate residues present in the C5aR N-terminus and important for C5a binding are well conserved across species (Supplementary Fig. S6). Furthermore, the second binding site in hC5aR, which is composed of charged residues on the extracellular membrane side such as Arg206 and of the hydrophobic pocket formed between helices H3 and H7 in the transmembrane region, is also well conserved between species (Supplementary Fig. S6) (Raffetseder *et al.*, 1996 ▶; Gerber *et al.*, 2001 ▶; Higginbottom *et al.*, 2005 ▶; Hagemann *et al.*, 2008 ▶). Recent data have suggested that the role of hC5a-desArg may have to be revisited (Reis *et al.*, 2012 ▶). Indeed, in a novel label-free cellular assay evaluating the global downstream cellular response to C5aR activation, physiological concentrations of hC5a-desArg could induce an overall cellular activation as high as, if not higher than, the intact hC5a anaphylatoxin (Reis *et al.*, 2012 ▶). Thus, the differential activation properties of these anaphylatoxins may also depend on the cell context and on the type of biological activity investigated. The sequence and geometry of the C5a C-terminus are also essential parameters in triggering the activation switch that conditions C5aR-mediated cellular response. Indeed, downstream G-protein coupling and activation seem to involve subtle changes in the relative orientation of C5aR helices H3 and H7 (Gerber *et al.*, 2001 ▶). For example, a single Ile116Ala substitution in the hydrophobic pocket of hC5aR constituting the second binding site for C5a transforms an antagonistic peptide mimicking the C5a C-terminus into an agonist. Inversely, changes in the C5a C-terminal topography will also have dramatic effects on C5aR activation. In this context, the structuring of hC5a-A8 into a β-strand motif extending outside the helix-bundle core could provide a mechanistic model for the two-step binding mode of hC5a to its receptor. Indeed, while the hC5a C-terminus may adopt the helical conformation of the NMR model (Zhang *et al.*, 1997 ▶) in solution and possibly keep this conformation throughout binding to the receptor, interaction of the hC5a core with its first binding site on the hC5aR N-terminus could also induce release of the hC5a C-terminus from the bundle core, and an extended conformation as observed for this region in hC5a-A8 may be required to allow the C-terminus to anchor into the transmembrane binding site on hC5aR. Nevertheless, it is not known whether such release is necessary and hence it remains difficult to speculate on precise mechanisms at this stage. Thus, deeper structural investigations, and notably a detailed atomic structure revealing the C5a–C5aR interaction, are required to gain detailed insights into C5aR activation by complement anaphylatoxins.

## 5. Related literature   

The following references are cited in the Supporting Information for this article: Bond & Schüttelkopf (2009 ▶), Bubeck *et al.* (1994 ▶), Chen *et al.* (1998 ▶), Cook *et al.* (2010 ▶), Corpet (1988 ▶), DeMartino *et al.* (1994 ▶), Farzan *et al.* (2001 ▶), Hagemann *et al.* (2006 ▶, 2008 ▶), McCoy *et al.* (2005 ▶), Mery & Boulay (1994 ▶), Mollison *et al.* (1989 ▶), Raffetseder *et al.* (1996 ▶), Siciliano *et al.* (1994 ▶) and Toth *et al.* (1994 ▶).

## Supplementary Material

Supplementary Figures S1-S6.. DOI: 10.1107/S139900471400844X/dw5099sup1.pdf

PDB reference: murine C5a, 4p3a

PDB reference: murine C5a-desArg, 4p3b

PDB reference: human C5a-A8^Δ71–73^, 4p39

## Figures and Tables

**Table 1:** Data-collection and refinement statistics for the hC5a-A8, mC5a and mC5a-desArg structures Values in parentheses are for the highest resolution shell.

	Human C5a-A8^7173^	Mouse C5a	Mouse C5a-desArg
Data-collection statistics			
X-ray source	X06SA, SLS	911-3, MAX-lab	911-3, MAX-lab
Wavelength ()	1.000	0.9792	1.000
Space group	*C*222_1_	*P*4_3_	*P*4_3_
Unit-cell parameters ()			
*a*	69.35	54.95	55.21
*b*	83.24	54.95	55.21
*c*	119.22	117.43	117.43
Resolution ()	502.4 (2.52.4)	501.4 (1.51.4)	502.1 (2.22.1)
Unique reflections	13812 (1541)	68172 (12739)	20489 (2655)
Completeness (%)	99.6 (98.5)	99.8 (99.8)	99.9 (100)
Multiplicity	6.2 (6.1)	4.2 (4.1)	6.4 (6.4)
*R*_meas_(*I*)† (%)	8.8 (60.4)	7.3 (84.9)	12.3 (72.2)
*I*/(*I*)	17.07 (3.81)	15.10 (2.33)	14.62 (2.95)
Mosaicity‡ ()	0.17	0.13	0.12
Wilson *B* ‡ (^2^)	60	20	30
Refinement statistics			
Resolution ()	492.4	371.4	332.1
Unique reflections	13432	68165	20350
*R*_work_ (%)	22.36	14.42	18.03
*R*_free_ (%)	23.77	17.40	22.41
Model in the asymmetric unit (No. of atoms)			
Protein	2143	2212	2212
Ligands		48	45
Water	22	333	204
Root-mean-square deviations			
Bonds ()	0.002	0.008	0.003
Angles ()	0.463	1.198	0.711
Average *B* values (^2^)			
Protein	68	19	35
Ligands	61	29	41
Ramachandran plot§ (%)			
Favoured	99.6	100	98.9
Allowed	0.4	0	1.1
Outliers	0	0	0

† *R*_meas_(*I*) = 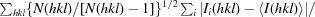 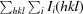.

‡ Value given by *XDS*.

§ Values given by *MolProbity*.

## Footnotes

1 Supporting information has been deposited in the IUCr electronic archive (Reference: DW5099).
