# Supplementary material for: Structural and functional characterization of human and murine C5a anaphylatoxins
Source: Acta Crystallogr D Biol Crystallogr. 2014 May 30;70(Pt 6):1704–17. doi: 10.1107/S139900471400844X (PMC4051506; doi:10.1107/S139900471400844X)
Supplement: Supplementary file 1 [file d-70-01704-sup1.pdf]

Supporting Information

Structural and functional characterization of human and murine C5a anaphylatoxins

Janus Asbjørn Schatz-Jakobsen[a], Laure Yatime[a], Casper Larsen[a], Steen Vang Petersen[b], Andreas Klos[c] and Gregers Rom Andersen[a]

[a] *Department of Molecular Biology and Genetics, Aarhus University, Gustav Wieds Vej 10C, DK-8000 Aarhus, Denmark.*

[b] *Department of Biomedicine, Aarhus University, Bartholin building, Wilhelm Meyers Allé 4, DK-8000 Aarhus, Denmark.*

[c] *Institute for Medical Microbiology and Hospital Epidemiology, Medical School Hannover, Hannover, Germany.*

CONTENT

Fig. S1 Sequence alignment for C5a proteins

Fig. S2 Mass spectrometry analysis of the recombinant C5a proteins

Fig. S3 Initial electron density maps for hC5a-A8 after MR using a three-helix bundle core model and final crystal packing in the hC5a-A8 structure

Fig. S4 Initial electron density maps for mC5a and mC5a-desArg after MR using a three-helix bundle core model

Fig. S5 Additional density in the mC5a structure possibly representating the C-terminal arginine residue

Fig. S6 Sequence alignment for C5a receptors (C5aR)

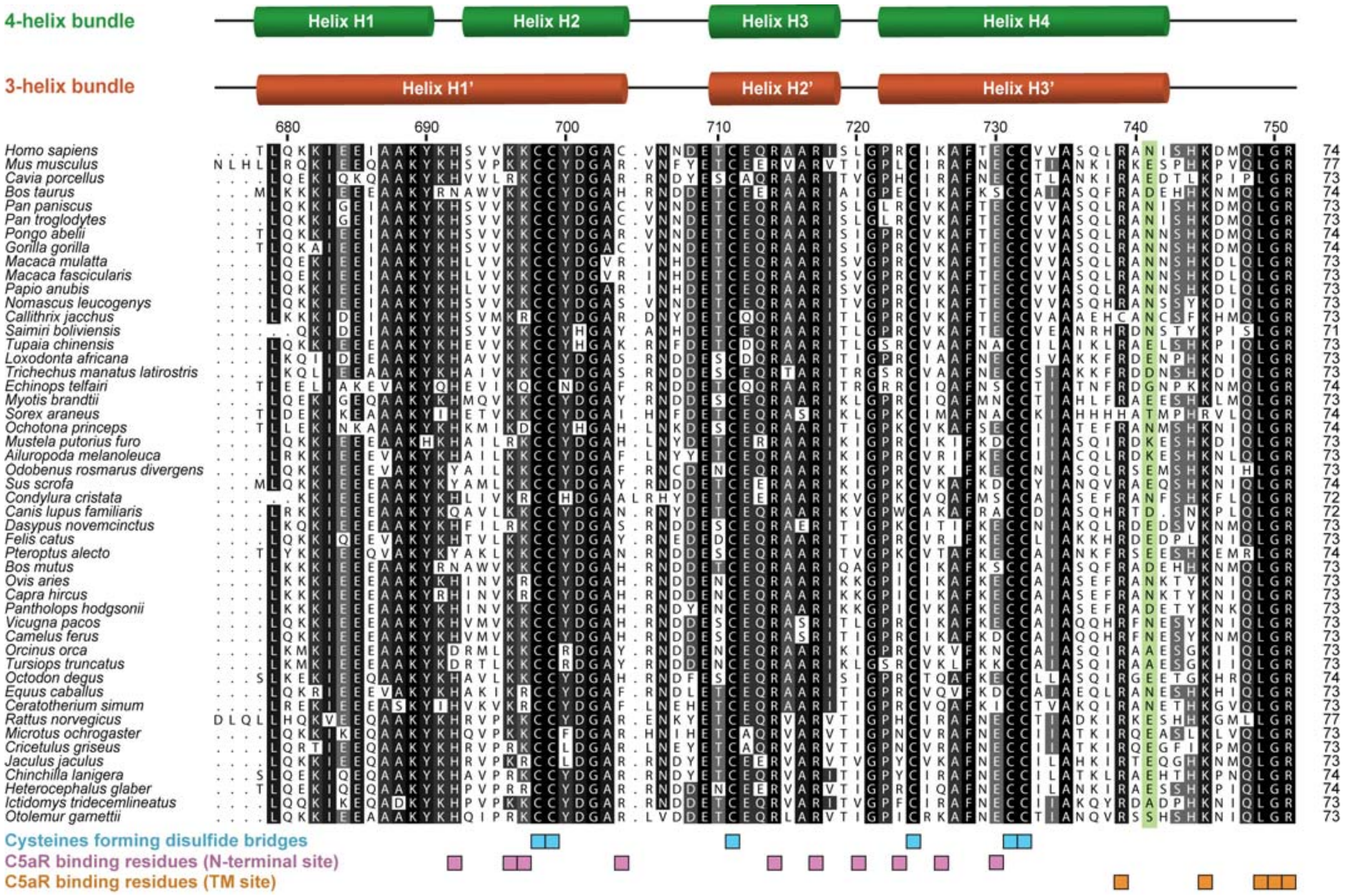

Fig. S1: Sequence alignment for C5a proteins. The sequences of forty-nine mammalian C5a proteins were aligned using MULTIALIN (Bond & Schuttelkopf, 2009) and the sequence conservation analysis was done in ALINE (Corpet, 1988). The position of the secondary structure elements in both the four- and three-helix bundle models are indicated above the alignment. The sequence numbering above the alignment refers to human C5 prepro-numbering, numbering according to C5a is obtained by subtracting 677. The glycosylated asparagine in human C5a is highlighted in green. Below the alignment are indicated cysteine residues involved in intramolecular disulfide bridges (blue) and residues identified as important for C5aR binding: C5a residues possibly involved in interactions with C5aR N-terminus are highlighted in purple and C5a residues presumably involved in docking C5a C-terminus in the transmembrane region of C5aR are shown in orange (Mollison *et al.*, 1989; Toth *et al.*, 1994; Bubeck *et al.*, 1994; Hagemann *et al.*, 2006; Hagemann *et al.*, 2008).

A

Protein	hC5a	hC5a-desArg	hC5a-A8	mC5a	mC5a-desArg
Theoretical M_w (Da) *	8792.15	8635.96	8344.64	9023.52	8867.33
M_w given by MS (Da)	8792.85	8636.39	8345.19	9023.66	8867.10

* assuming 6 cysteines engaged in a disulfide bridge per protein

B

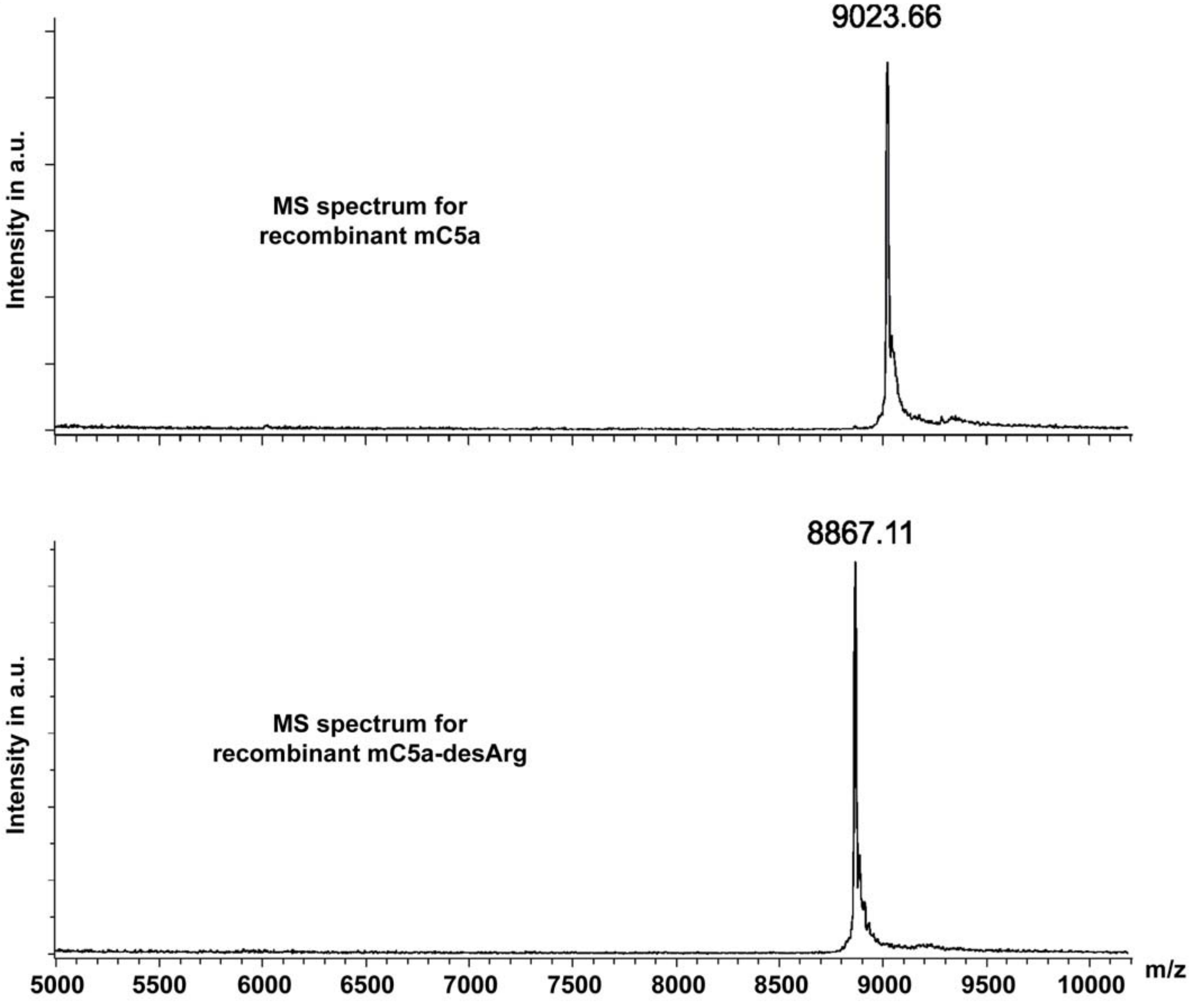

Fig. S2: Mass spectrometry analysis of all recombinant C5a proteins produced in this study. A) Comparison between the molecular masses obtained by MS analysis and the theoretical values expected for the different recombinant C5a proteins. **B)** Examples of MS spectra obtained for recombinant mC5a and mC5a-desArg.

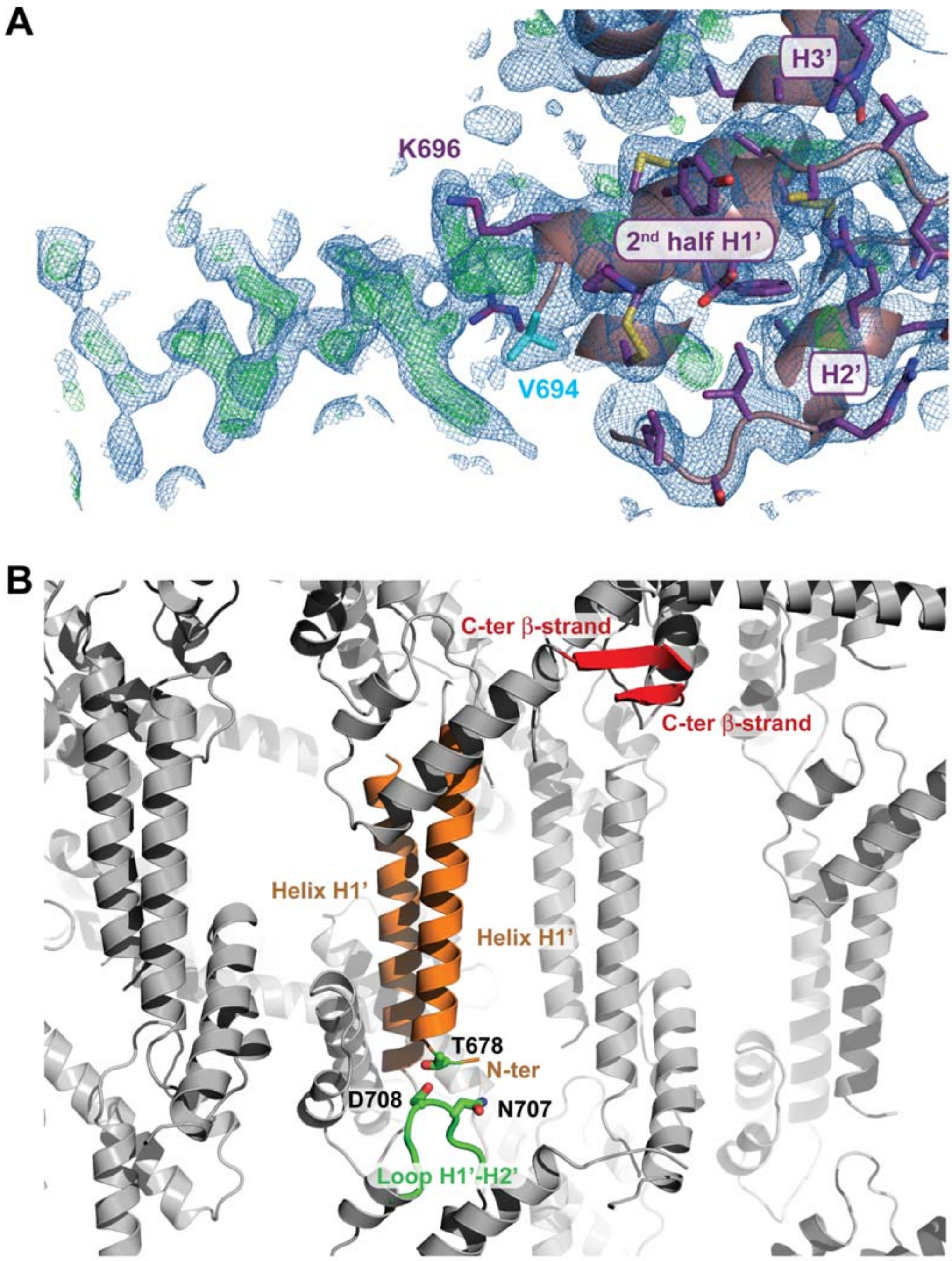

Fig. S3: Initial electron density maps and crystal packing in hC5a-A8 structure. A) Initial electron density maps obtained after molecular replacement in PHASER (McCoy *et al.*, 2005) using a MR search model derived from hC5a-desArg, encompassing residues Val694-Ser743 of monomer A from RCSB entry 3HQA (Cook *et al.*, 2010). The $2mF_o$-DF_c map is displayed as blue mesh and contoured at 1σ. The mF_o-DF_c map is displayed as green mesh and contoured at 2.5σ. **B)** The crystal packing in the hC5a-A8 structure is stabilized by three major types of interactions: the antiparallel packing of two helices H1' from neighbouring molecules (orange), the interaction of helix H1' N-terminus with the H1'-H2' loop from a symmetry-related molecule (green), and the formation of an antiparallel, two-stranded β-sheet between the C-termini of two hC5a-A8 molecules (red).

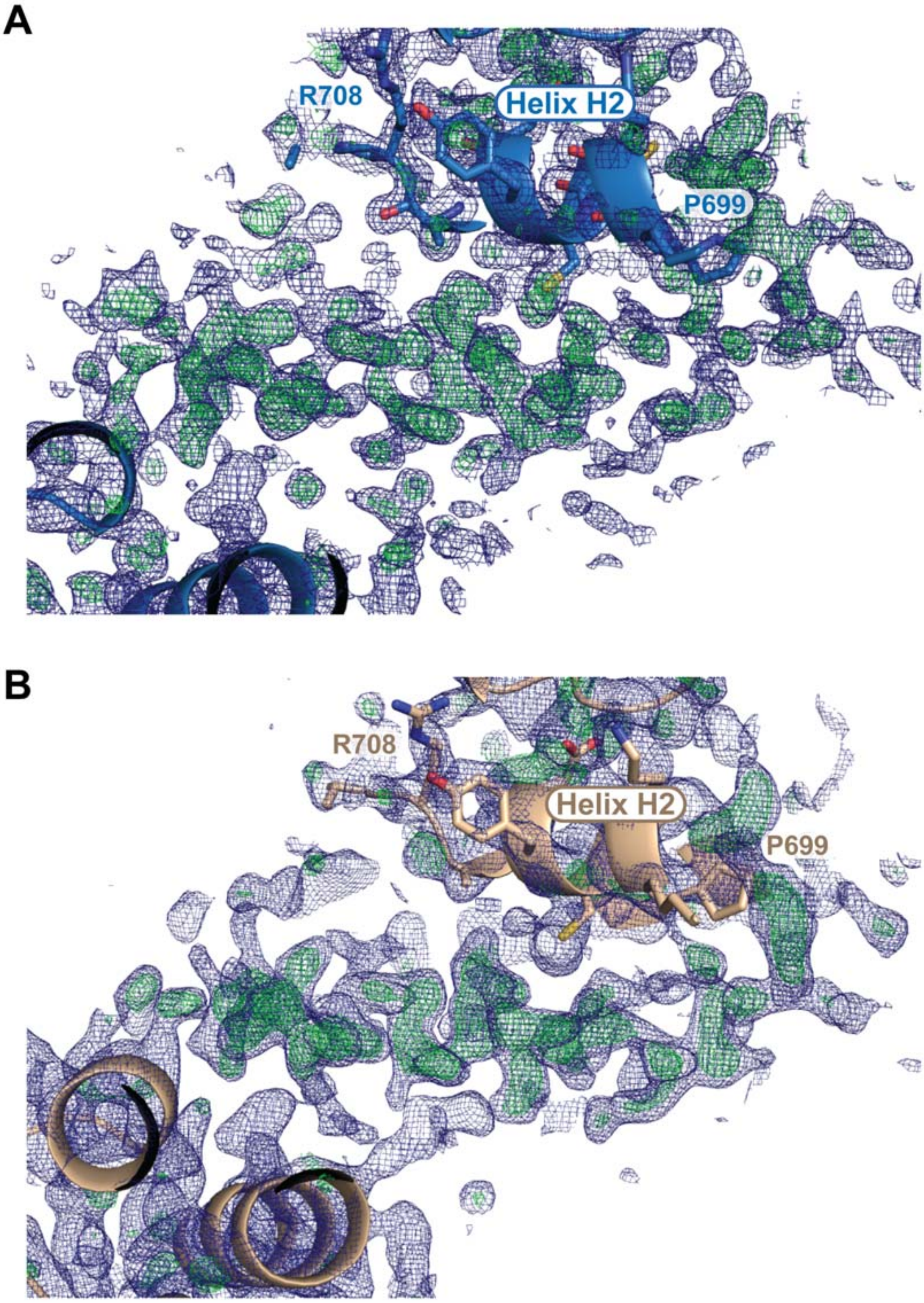

Fig. S4: Initial electron density maps for mC5a and mC5a-desArg. A) Initial electron density maps obtained after molecular replacement in PHASER (McCoy *et al.*, 2005) using a mC5a homology model derived from hC5a-desArg, encompassing residues Val694-Ser743 of monomer A from RCSB entry 3HQA (Cook *et al.*, 2010). The $2mF_o$-DF_c map is displayed as blue mesh and contoured at 1σ. The mF_o-DF_c map is displayed as green mesh and contoured at 2.5σ. Clear density for helix H1 not part of the search model is observed in the middle of the panel. **B)** Same as panel A but for the mC5a-desArg structure.

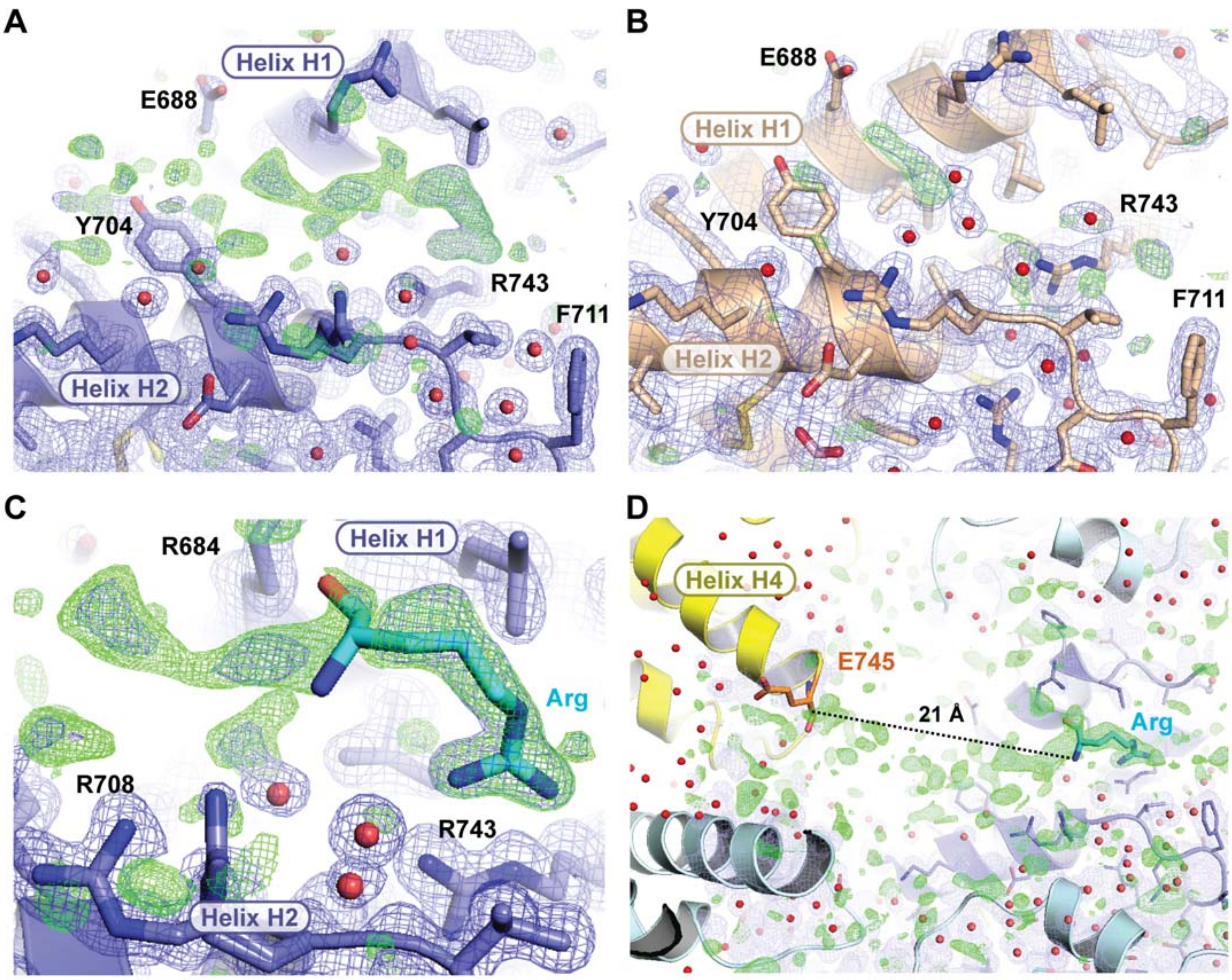

Fig. S5: Additional density possibly representing the C-terminal arginine residue in mC5a structure. A) Final 2mF_o-DF_c (blue mesh) and mF_o-DF_c (green mesh) maps contoured at 1 and 3σ, respectively, for the mC5a structure. Clear additional density assuming the shape of an arginine, and not accounted for by water molecules or ligands, is visible in the groove between helices H1 and H2. Additional density extending away from this blob along the H1-H2 groove is also visible. **B)** Same as in panel A but for the mC5a-desArg structure. No similar additional density as for mC5a is visible here. **C)** Modeling of an arginine residue into the density observed in the mC5a structure reveals a quite reasonable fit. **D)** Larger view on the crystal packing in mC5a crystal revealing that this putative C-terminal arginine residue could arise from a symmetry-related molecule (displayed in yellow). The last modeled residue for that molecule, Glu745, is separated by 21 Å from the putative Arg and there are visible blobs of density in the solvent pocket separating the two residues.

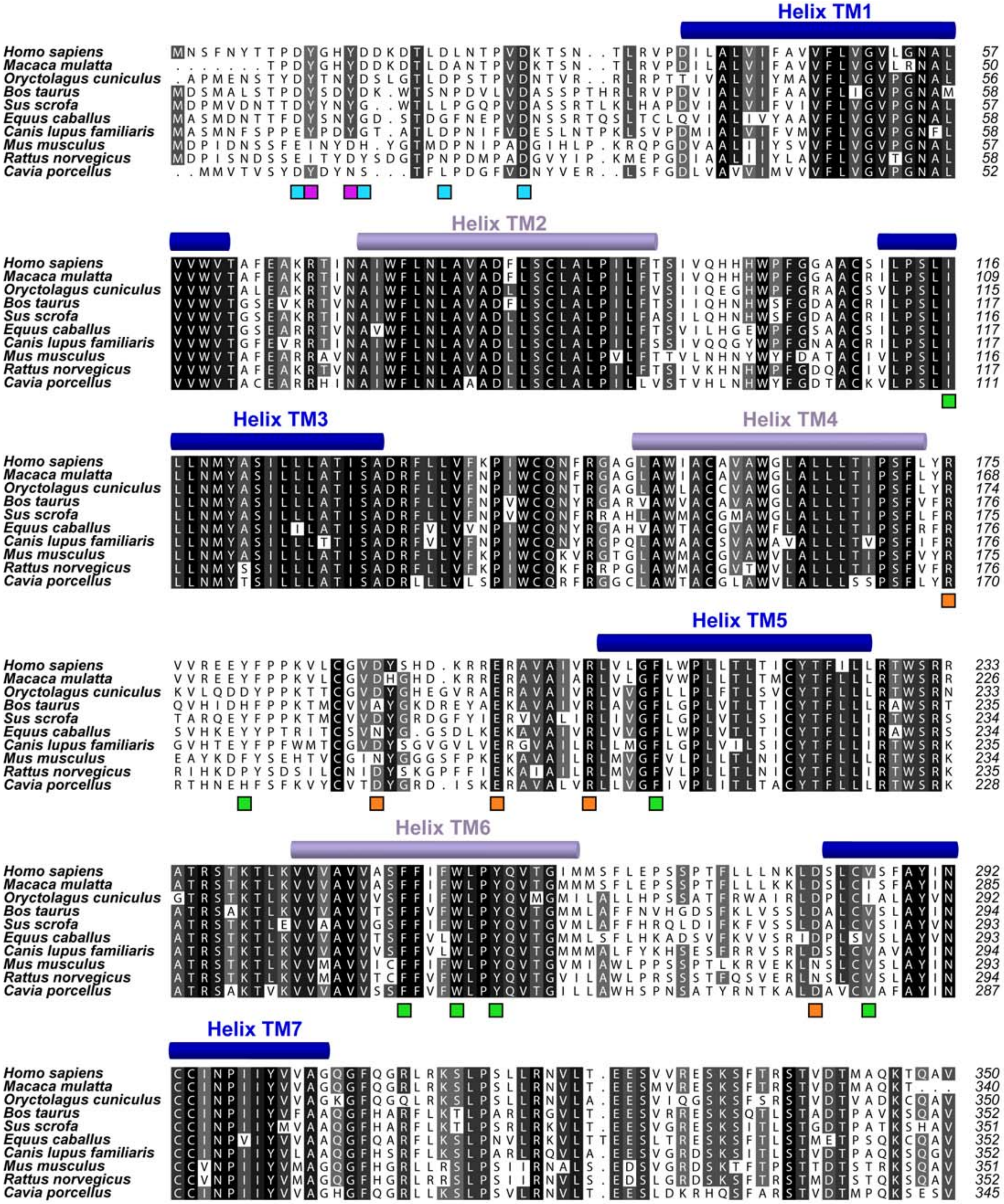
Helix TM1
Helix TM2
Helix TM3
Helix TM4
Helix TM5
Helix TM6
Helix TM7
Homo sapiens
Macaca mulatta
Oryctolagus cuniculus
Bos taurus
Sus scrofa
Equus caballus
Canis lupus familiaris
Mus musculus
Rattus norvegicus
Cavia porcellus
57 50 56 58 57 58 58 57 58 52
116 109 115 117 116 117 117 116 117 111
175 168 174 176 175 176 176 175 176 170
233 226 233 235 234 234 235 234 235 228
292 285 292 294 293 293 294 293 294 287
350 340 350 352 351 352 352 351 352 345

Fig. S6: Sequence alignment for C5aR proteins. The sequences of ten mammalian C5aR proteins were aligned using MULTIALIN (Bond & Schuttelkopf, 2009) and the sequence conservation analysis was performed in ALINE (Corpet, 1988). The putative positions of the seven transmembrane helices are indicated above the alignment. Below the alignment are indicated residues identified as important for C5a binding: sulfo-tyrosines (purple) and aspartate residues (cyan) from C5aR N-terminus supposedly interacting with C5a core, and charged (orange) and hydrophobic residues (green) supposedly involved in docking C5a C-terminus in a transmembrane pocket within C5aR (DeMartino *et al.*, 1994; Siciliano *et al.*, 1994; Chen *et al.*, 1998; Mery & Boulay, 1994; Raffetseder *et al.*, 1996; Farzan *et al.*, 2001; Hagemann *et al.*, 2008).

References

Bond, C. S. & Schuttelkopf, A. W. (2009). *Acta Cryst. D* 65, 510-512.

Bubeck, P., Grotzinger, J., Winkler, M., Kohl, J., Wollmer, A., Klos, A. & Bautsch, W. (1994). *Eur. J. Biochem.* 219, 897-904.

Chen, Z., Zhang, X., Gonnella, N. C., Pellas, T. C., Boyar, W. C. & Ni, F. (1998). *J. Biol. Chem.* 273, 10411-10419.

Cook, W. J., Galakatos, N., Boyar, W. C., Walter, R. L. & Ealick, S. E. (2010). *Acta Cryst. D* 66, 190-197.

Corpet, F. (1988). *Nucleic Acids Res.* 16, 10881-10890.

DeMartino, J. A., Van Riper, G., Siciliano, S. J., Molineaux, C. J., Konteatis, Z. D., Rosen, H. & Springer, M. S. (1994). *J. Biol. Chem.* 269, 14446-14450.

Farzan, M., Schnitzler, C. E., Vasilieva, N., Leung, D., Kuhn, J., Gerard, C., Gerard, N. P. & Choe, H. (2001). *J. Exp. Med.* 193, 1059-1066.

Hagemann, I. S., Miller, D. L., Klco, J. M., Nikiforovich, G. V. & Baranski, T. J. (2008). *J. Biol. Chem.* 283, 7763-7775.

Hagemann, I. S., Narzinski, K. D., Floyd, D. H. & Baranski, T. J. (2006). *J. Biol. Chem.* 281, 36783-36792.

McCoy, A. J., Grosse-Kunstleve, R. W., Storoni, L. C. & Read, R. J. (2005). *Acta Cryst. D* 61, 458-464.

Mery, L. & Boulay, F. (1994). *J. Biol. Chem.* 269, 3457-3463.

Mollison, K. W., Mandecki, W., Zuiderweg, E. R., Fayer, L., Fey, T. A., Krause, R. A., Conway, R. G., Miller, L., Edalji, R. P., Shallcross, M. A. & et al. (1989). *Proc. Natl. Acad. Sci. U. S. A.* 86, 292-296.

Raffetseder, U., Roper, D., Mery, L., Gietz, C., Klos, A., Grotzinger, J., Wollmer, A., Boulay, F., Kohl, J. & Bautsch, W. (1996). *Eur. J. Biochem.* 235, 82-90.

Siciliano, S. J., Rollins, T. E., DeMartino, J., Konteatis, Z., Malkowitz, L., Van Riper, G., Bondy, S., Rosen, H. & Springer, M. S. (1994). *Proc. Natl. Acad. Sci. U. S. A.* 91, 1214-1218.

Toth, M. J., Huwyler, L., Boyar, W. C., Braunwalder, A. F., Yarwood, D., Hadala, J., Haston, W. O., Sills, M. A., Seligmann, B. & Galakatos, N. (1994). *Protein Sci.* 3, 1159-1168.
